# Surgical approaches to canine appendicular osteosarcoma part 2 – limb-sparing techniques

**DOI:** 10.3389/fvets.2025.1655874

**Published:** 2025-12-17

**Authors:** Ryshely Sonaly de Moura Borges, Paloma Helena Sanches da Silva, Pedro Antônio Bronhara Pimentel, Renato Pereira Dornas, Angel Almendros, Antonio Giuliano, Rodrigo dos Santos Horta, Paulo Vinícius Tertuliano Marinho

**Affiliations:** 1 Department of Veterinary Medicine and Surgery, Veterinary School, Universidade Federal de Minas Gerais, Belo Horizonte, Brazil; 2 Department of Veterinary Clinical Sciences, Jockey Club College of Veterinary Medicine, City University of Hong Kong, Kowloon, Hong Kong SAR, China; 3 Harvest Veterinary Oncology Center, Kowloon, Hong Kong SAR, China; 4 Department of Veterinary Medicine, Instituto Federal Sul de Minas Gerais, Muzambinho, Brazil

**Keywords:** appendicular skeleton, bone tumor, oncological surgery, oncology, surgical anatomy

## Abstract

Osteosarcoma (OSA) is one of the main malignant primary bone neoplasms affecting humans and other vertebrate animals, and it represents the most common bone tumor in dogs, mainly affecting the appendicular skeleton. Given the highly aggressive nature of this cancer and the poor prognosis, immediate surgical intervention is recommended to achieve local control. Surgical treatment options may include radical surgery of the affected limb, considered the standard procedure, or limb preservation in selected cases. The purpose of this narrative literature review is to describe the limb-sparing techniques performed in the treatment of canine appendicular OSA. Limb-preserving techniques may include partial or total scapulectomy, excision of the bone segment with the tumor, and reconstruction using cortical allografts or metal endoprosthesis. Other options may involve endoexoprosthesis, pausterized tumor autografts, roll-over transposition of the ulna, limb shortening, and distraction due to bone transport. Those techniques are satisfactory in maintaining quality of life and may offer a good local disease control if the patient is properly selected, usually at initial stage.

## 1 Introduction

Osteosarcoma (OSA) is the most prevalent primary bone cancer in dogs, particularly affecting the appendicular skeleton (1, 2). Due to the aggressive nature of this malignancy and the generally poor prognosis, prompt surgical treatment is recommended for effective local control (3).

In addition to more radical surgeries involving limb amputation, other surgical treatment options can be used, such as limb-sparing surgeries, including partial or total scapulectomy and partial ulnectomy, as well as reconstructive procedures (3–6). Limb-sparing techniques that prioritize tumor resection with appropriate surgical margins require rigorous patient selection criteria and meticulous surgical planning. These approaches must ensure both adequate limb functionality and effective local disease control (7).

In summary, surgical treatment of canine appendicular OSA requires essential prerequisites beyond the patient’s general condition, including knowledge of the biological behavior of the tumor and a thorough understanding of surgical techniques. These techniques should be based on the topographic anatomy of the bones, joints, ligaments, tendons, muscles, blood vessels, and nerves within the limb to avoid unnecessary dissection and transection. Thus, this article aims to describe surgical approaches related to limb preservation techniques for the treatment of appendicular OSA in dogs, based on current best practices and evidence, with particular emphasis on relevant anatomical considerations.

## 2 Limb-sparing techniques

Limb-sparing procedures include partial or total scapulectomy (8, 9), partial ulnectomy, bone segment excision followed by reconstruction using cortical allografts, metallic endoprostheses (5), endo-exoprostheses (10), pasteurized tumor autografts (11), ulnar rollover transposition (6), limb shortening, or bone transport distraction.

Limb-sparing surgeries are useful when tumor resection compromises the functional integrity of the limb and are typically performed in anatomically challenging locations (12). They are indicated for patients who are not candidates for amputation due to concomitant orthopedic or neurological conditions, a history of prior amputation, or in giant breeds (16), with survival rates comparable to those of amputations (13).

In practice, limb-sparing techniques are more effective for tumors in the distal radius compared to the pelvic limb and other regions of the thoracic limb. Consequently, they are more frequently performed and offer more treatment options for this location. Llido et al. (14) states that, at present, no limb-sparing options exist for proximal humeral OSA with an acceptable risk of complications. Ambulation following pancarpal arthrodesis is generally favorable, unlike cases involving tumors in the shoulder, stifle, or tarsal joints (15, 16).

Candidates for limb-sparing surgery include dogs with appendicular OSA without metastasis, with less than 50% of the bone length affected, minimal soft tissue involvement, and lesions that do not extend distally beyond the antebrachiocarpal joint. Additionally, candidates should have good cardiac, renal, and bone marrow function, as well as intact skin. Limb-sparing is considered for patients who are not suitable for amputation due to a low likelihood of regaining limb function, severe orthopedic or neurological comorbidities, or owner refusal (13, 15–18).

This technique is disregarded in patients with compromised surrounding soft tissues, particularly in cases of circumferential (360°) involvement. Pathological fractures are a relative contraindication due to the increased risk of local recurrence, as tumor cells may spread to adjacent tissues (17). Overall, complication rates associated with limb-sparing techniques are higher than those observed with amputation (57).

The assessment of tumor extent in bone tissue, similar to patients undergoing amputations, can be performed using radiography, nuclear scintigraphy, CT, or magnetic resonance imaging (MRI) (56). Bone margins should remain at least 3 cm, and 2–3 cm in muscle layers and other soft tissues (15). Radiography may overestimate the proximal extent of distal radial OSA lesions in dogs (19). Wallack et al. (20) described that in lateromedial projections, the overestimation is 17 ± 28% and in craniocaudal projections it was 4 ± 26%. Cruz et al. (21) concluded that the average radiographic overestimation in mediolateral projections is 9.29%. CT overestimates the intramedullary extension of tumors, with an average of 1.5% overestimation of tumor length, reaching up to 27% (22). Meanwhile, MRI presents a 3% overestimation of intramedullary tumor involvement when evaluated in T1-weighted images (20). Although tumor overestimation may potentially reduce the risk of incomplete excision, it also increases the chance of excluding patients eligible for limb-sparing surgery (19).

Various techniques are available for treating distal radial OSA in dogs. Among them, cortical allograft, pasteurized autograft, endoprosthesis, vascularized ulnar transposition (ulnar rollover), and bone transport osteogenesis require prior resection of the distal radius. Stereotactic radiosurgery, on the other hand, does not require distal radius resection. Each of these techniques has its own advantages and potential complications, making careful and individualized patient evaluation essential.

### 2.1 Scapulectomy

Scapulectomy involves the surgical removal of the scapula and can be performed as partial, subtotal, or total, depending on the extent of the lesion (8, 15, 23, 24, 57). This technique is indicated for scapular neoplasms that do not involve adjacent soft tissues, provided a minimum distal margin of 3 cm can be achieved to ensure oncological adequacy (8, 15, 24, 57). Despite these indications, scapulectomy is infrequently performed in clinical practice, as malignant tumors affecting the scapula are rare in dogs (8, 25).

In a retrospective study of 42 dogs with scapular neoplasia, OSA was the most prevalent tumor, diagnosed in 64.5% of cases and managed via subtotal scapulectomy, which preserved thoracic limb function (8, 9). All three surgical approaches to the scapula (partial, subtotal, or total) can maintain a viable and functional thoracic limb, provided the superficial and deep scapular muscles are preserved or reconstructed. Failure to restore these muscles results in instability and loss of limb support during movement (8). When soft tissue reconstruction is performed effectively, endoprosthesis implantation is unnecessary, making scapulectomy a viable alternative to high thoracic limb amputation. This technique achieves wide surgical margins while sparing the limb, provided the tumor does not extend into adjacent tissues (8, 9, 24).

Partial scapulectomy involves resection of up to three-quarters of the scapula while preserving the acromion, the acromial head of the *deltoid* muscle, and the distal portions of the *infraspinatus* and *supraspinatus* muscles. This technique enhances scapulohumeral joint stability by excising the proximal scapula. Subtotal scapulectomy removes more than three-quarters of the bone, extending to the scapular notch, while preserving the glenoid cavity and shoulder joint. Total scapulectomy, which entails complete scapular removal via glenohumeral disarticulation, is reserved for distal scapular involvement or cases where adequate surgical margins cannot otherwise be achieved (8, 26, 57). Potential complications include limb fixation failure, pneumothorax, and surgical site seroma (8, 26).

**Figure 1:**
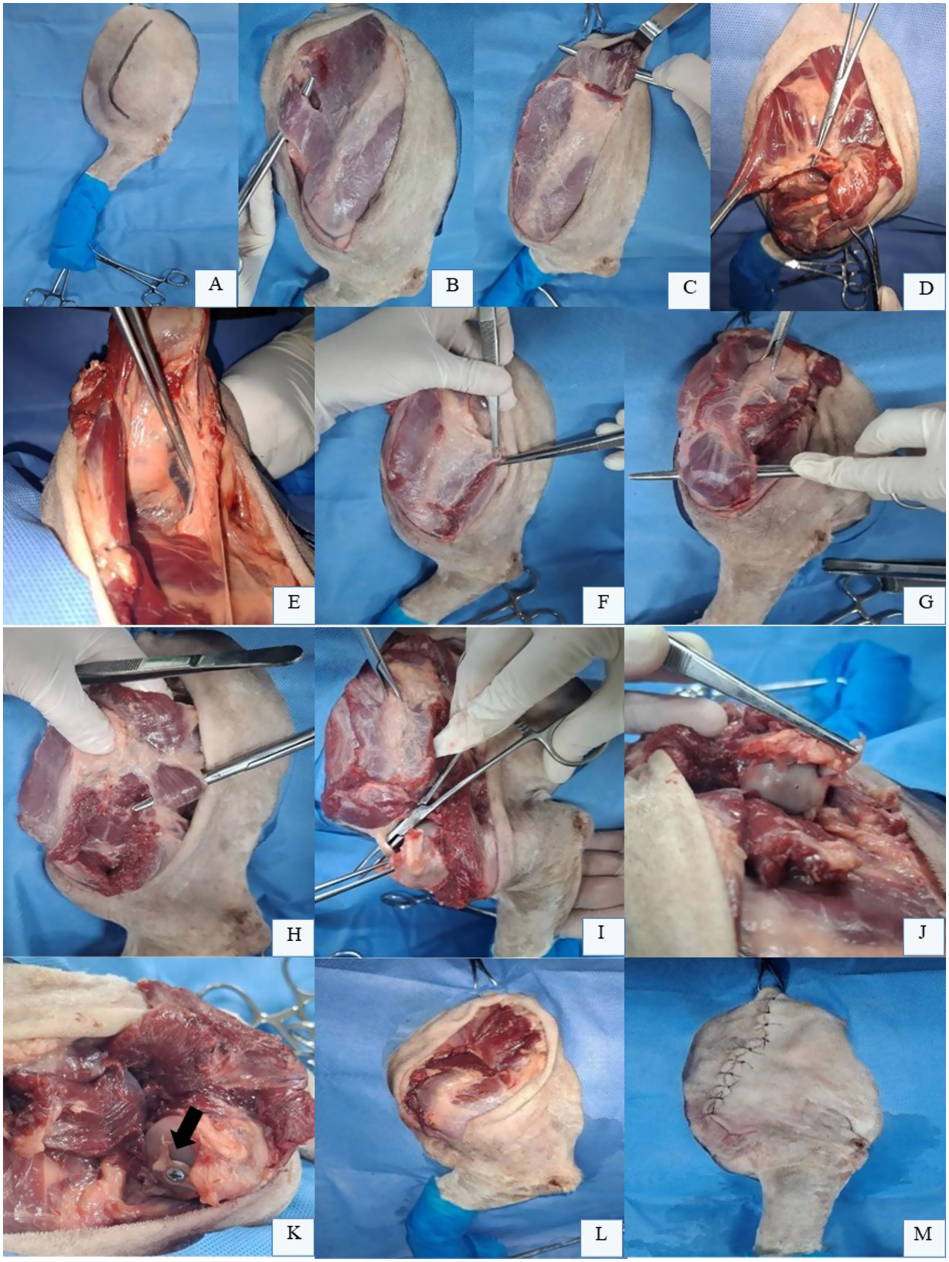
Total scapulectomy in the left thoracic limb (LTL) of a canine cadaver positioned in right lateral recumbency. **(A)** Skin incision marked along the scapular spine, extending to the mid-third of the humerus. **(B)** Reflected skin and subcutaneous tissue expose the *omotransversarius* muscle (isolated over forceps) for transection 3.0 cm from its scapular spine insertion. **(C)** *Rhomboideus* muscle (over forceps) isolated and transected along the dorsal scapular border. **(D)** *Serratus ventralis* muscle (grasped by tissue forceps) elevated from the serrated scapular face during lateral traction with a Backhaus clamp. Note the lateral cutaneous branch requiring ligation for hemostasis. **(E)** Laterally retracted scapula revealing the axillary vein and artery (Debakey forceps) and brachial plexus (within adipose tissue), which are preserved. **(F)** Scapular portion of the *deltoideus* muscle transected with a safety margin. **(G)** Acromial portion of the *deltoideus* muscle isolated for transection with a safety margin. **(H)** Isolation and transection of the *teres major* muscle (over forceps) and long head of the *triceps brachii* (black arrow) at the caudal scapular border. **(I)** Tendons of the *supraspinatus* (clamped), *infraspinatus*, *coracobrachialis*, *teres minor*, and *subscapularis* muscles transected from the humeral head. **(J)** *Biceps brachii* tendon grasped with tissue forceps. **(K)** *Biceps brachii* tenodesis to the humeral head using a bone screw (black arrow). **(L)** Myorrhaphy of preserved muscles over the humeral head. **(M)** Final surgical site appearance after total scapulectomy. Original images captured by the authors during a cadaveric surgical demonstration for academic purposes; none of the images have been published in any other scientific work.

For total scapulectomy, the surgical field is clipped from the dorsal midline to below the elbow joint, followed by aseptic preparation. The patient is positioned in lateral recumbency with the affected limb uppermost. The initial approach begins over the scapular spine. A skin incision is made along the dorsal aspect of the scapular spine, extending to the mid-third of the humerus (Figure 1A). The skin and subcutaneous tissue overlying the scapular region are preserved and reflected. With the limb adducted, the *omotransversarius* muscle (Figure 1B) and cervical portion of the *trapezius* muscle are identified superficially and cranially to the scapular spine. These muscles are isolated and transected 3 cm from their scapular insertions to maintain adequate surgical margins. The thoracic portion of the *trapezius* muscle and the cervical/thoracic parts of the *rhomboideus* muscle along the dorsal scapular border are similarly transected (Figure 1C).

Transection of the *rhomboideus* muscle exposes the underlying serratus ventralis muscle. Using an osteotome, the *serratus ventralis* muscle is elevated from the costal surface of the scapula, facilitated by medial scapular exposure achieved with a Backhaus clamp applying lateral traction (Figure 1D). A lateral cutaneous branch within the *serratus ventralis* muscle is identified and double-ligated with absorbable monofilament suture for hemostasis. Care should be taken to preserve the axillary vessels and brachial plexus during medial scapular dissection (Figure 1E). With the limb readducted, the scapular (Figure 1F) and acromial portions (Figure 1G) of the *deltoideus* muscle are transected, along with the insertions of the *teres major* muscle and long head of the *triceps brachii* at the caudal scapular border (Figure 1H). The tendons of the *supraspinatus*, *infraspinatus*, *coracobrachialis*, *teres minor*, and *subscapularis* muscles are transected from the humeral head (Figure 1I). The suprascapular and subscapular nerves are infiltrated with local anesthetic (e.g., 2 mg/kg bupivacaine) and may be transected (58).

To complete scapular removal, the joint capsule is incised for glenohumeral disarticulation. The tendon of the *biceps brachii* muscle is then transected from its origin at the supraglenoid tubercle (Figure 1J) and may be sutured to the residual joint capsule on the proximal humerus using an interrupted horizontal mattress or Mayo mattress sutures with 2–0 non-absorbable suture.

Limb function may also be enhanced through *biceps brachii* tenodesis to the humeral head using a bone screw (Figure 1K), as this muscle is critical for shoulder extension, elbow flexion, and joint stabilization (25). Additionally, suturing of all preserved musculature is necessary to provide structural support to the thoracic limb (Figure 1L) (8). The long head of the *triceps brachii* is sutured to the *omotransversarius* and trapezius muscles at the humeral head. The *trapezius* is then sutured to the *serratus ventralis* muscle and the scapular portion of the *deltoideus* muscle is superficially apposed to the *transversus* muscle. Finally, dead space obliteration and skin closure are performed (Figure 1M) (8, 15, 57).

**Figure 2:**
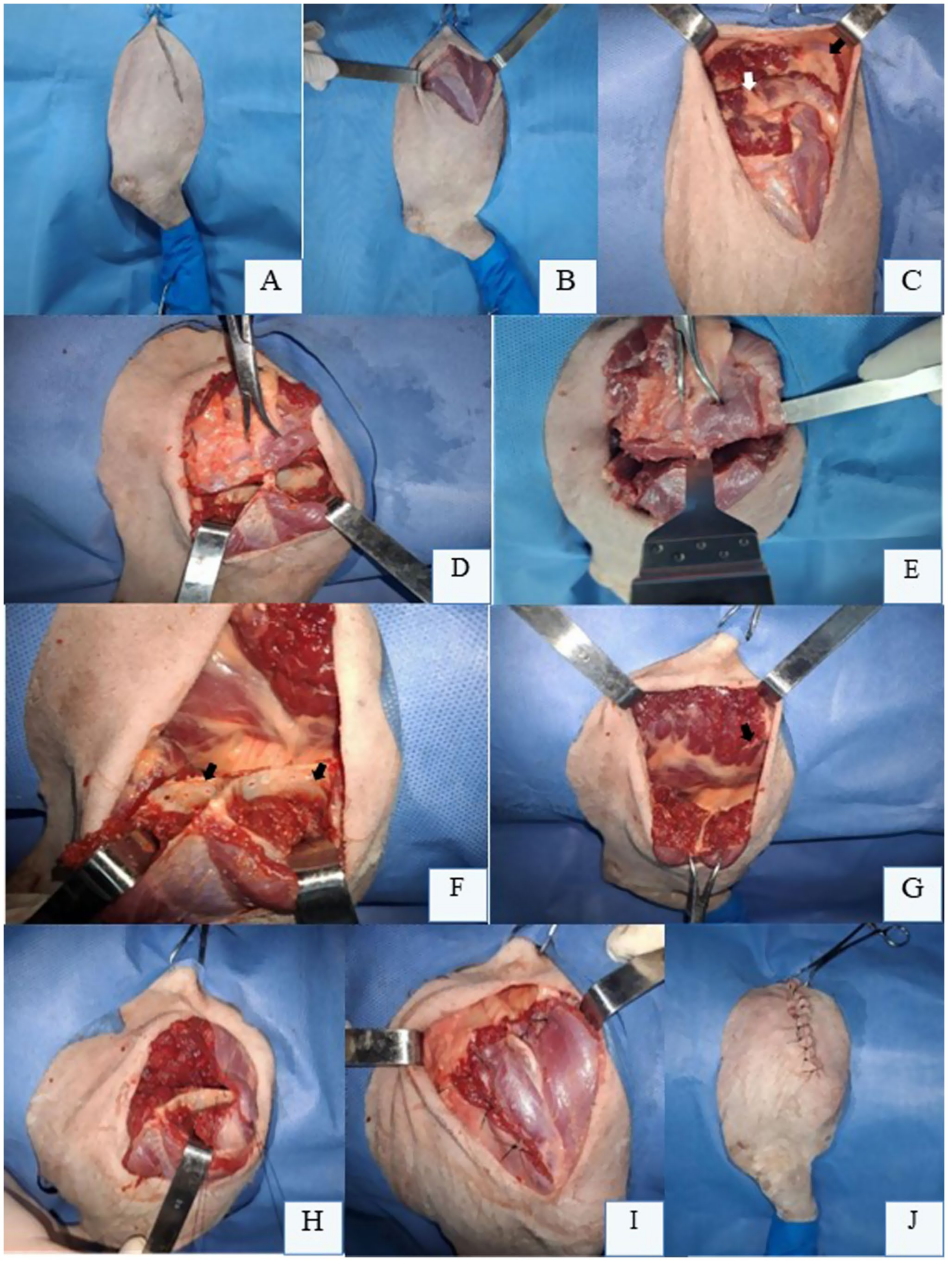
Partial scapulectomy in a canine cadaver’s right thoracic limb (RTL) positioned in left lateral recumbency. **(A)** Demarcation line for skin incision over the scapular spine. **(B)** After skin and subcutaneous tissue reflection, the extrinsic muscles of the scapula are exposed for transection, maintaining a 3 cm margin from their respective insertions on the scapular spine. **(C)** Transection of the *trapezius* muscle (black arrow) and *rhomboideus* muscle (white arrow) to release the dorsal aspect of the scapula. **(D)** Retraction of the *supraspinatus* (black arrow) and *infraspinatus* (white arrow) muscles caudally using Farabeuf retractors after their incision and fiber separation with an osteotome at the intended osteotomy site. **(E)** Bone saw positioned perpendicularly to the scapular spine and fossae for partial scapulectomy. **(F)** Drilled holes (black arrow) along the proximal border of the remaining scapula for muscle anchoring. **(G)** Scapula appearance after osteotomy at its middle third, with the remaining *serratus ventralis* (black arrow) and *rhomboideus* (white arrow) muscles visible. **(H)** Muscle anchoring to the proximal border of the residual scapula using non-absorbable suture in an interrupted cruciate suture pattern. **(I)** Final appearance of the myorrhaphy between the remaining extrinsic scapular muscles and the *supraspinatus* and *infraspinatus* muscles following partial scapular removal. **(J)** Skin closure and final appearance of the RTL after partial scapulectomy. Original images captured by the authors during a cadaveric surgical demonstration for academic purposes; none of the images have been published in any other scientific work.

For partial scapulectomy, the initial approach mirrors total scapulectomy, with the patient positioned in lateral recumbency and a skin incision confined to the scapular spine (Figure 2A). For proximal scapular lesions, 3 cm safety margins are established. The extrinsic scapular muscles—cervical and thoracic portions of the *trapezius* muscle (Figure 2B)—are isolated and transected lateral to the scapular spine, though the *omotransversarius* muscle may be spared distally if margins permit. The *rhomboideus* muscle (cervical and thoracic portions) is transected along the dorsal scapular border, followed by elevation of the *serratus ventralis* muscle from the medial scapular surface (Figure 2C).

With the scapula dorsally freed, the *supraspinatus* and *infraspinatus* muscles are transected with a scalpel, maintaining appropriate margins, while an osteotome aids in retracting muscle fibers from the *supraspinous* and *infraspinous* fossae (Figure 2D). A bone saw is then positioned perpendicularly across these fossae and the scapular spine for osteotomy (Figure 2E). Small drill holes are created in the proximal remnant scapula using a fine burr and low-speed drill (Figure 2F). The *serratus ventralis* (Figure 2G), *rhomboideus*, and preserved portions of the *trapezius* muscle are anchored to these holes with heavy-gauge non-absorbable suture, replicating their original scapular insertions (Figure 2H). Anchoring begins with deep muscles, progressing superficially (Figure 2I). Dead space is meticulously obliterated during subcutaneous closure to prevent seroma; a closed suction drain may be employed if necessary. Routine intradermal or cutaneous sutures complete the procedure (Figure 2J).

If subtotal scapulectomy is required, the technique is similar, but the osteotomy is performed either proximally or at the level of the scapular notch or supraglenoid tubercle (8, 9). This approach preserves the glenoid cavity and the origin of the *biceps brachii* tendon, while the muscle edges of the *supraspinatus*, *infraspinatus*, *deltoid*, and long head of the *triceps* are sutured to the *serratus ventralis*, *omotransversarius*, and *trapezius* muscles. This closure fills the defect area, helping to minimize dorsal displacement of the remaining scapular segment during the stance phase of locomotion.

Postoperative complications are uncommon, with the most frequently reported being lameness, which tends to improve over time, and seroma formation (8, 15, 16). In dogs, postoperative limb use quality has been negatively correlated with increased body weight but is not influenced by the extent of scapular excision (8).

In a retrospective study, Montinaro et al. (8) described a series of 42 dogs diagnosed with scapular OSA and treated with scapulectomy. The primary clinical complaint was lameness, observed in 86% of patients. Partial scapulectomy was performed in 24 dogs, subtotal in 13, and total in 5. In 81% of cases, more than 50% of the scapula was removed. The percentage of scapula excised was not associated with postoperative limb use, disease-free interval, or survival time. The reported disease-free interval was 204 days, and the median survival time was 246 days. The complication rate following scapulectomy was low, with seroma occurring in seven dogs and one case of incisional dehiscence at the level of the *serratus ventralis* muscle (8).

For resection of the distal radius portion, the thoracic limb should be clipped and aseptically prepared rigorously, from the digits to the shoulder joint, in case the procedure needs to be converted into an amputation. Intravenous prophylactic antibiotics should be administered, and due to the high infection rates reported, the surgical team, the preparation of the operating room, and the flow of personnel should be carefully controlled (15, 27).

The patient may be placed in a lateral recumbency position, with the affected limb facing upward, dorsal recumbency with the limb pulled caudally, or sternal recumbency with the limb pulled cranially, depending on the approach, which can be cranial, craniolateral, or craniomedial. Make an incision that extends from the distal region to the elbow joint, up to above the third or fourth metacarpal bone. Proceed with the dissection of the subcutaneous tissue and antebrachial fascia, preserving the cephalic vein when possible (15, 57).

In the craniolateral approach, the deep antebrachial fascia is incised between the *extensor carpi radialis* and common digital extensors muscles, and these structures are then retracted to expose part of the radial diaphysis. Functional preservation of the extensor muscles and tendons is not mandatory, but care should be taken to keep the radial nerve intact. The *supinator* and p*ronator* muscles are elevated from the proximal part of the radius using a periosteal elevator. A combination of sharp and blunt dissection is employed outside the tumor pseudocapsule, which is typically identified by abnormal tissue coloration and hypervascularity. The *abductor pollicis longus*, *extensor carpi radialis*, common digital extensor, lateral digital extensor, and lateral ulnar muscles are resected at the osteotomy level. The transection of the extensor muscles can be performed without compromising the limb’s function; however, preservation of the lateral ulnar muscle is recommended when feasible to maintain soft tissue coverage around the implant (15, 57).

A radial osteotomy is performed at least 3 cm proximal to the tumor’s proximal extent, with en bloc excision of soft tissues involved in the neoplastic capsule, maintaining equivalent margins. A bone marrow sample from the proximal osteotomy site should be collected for histologic evaluation to confirm margin adequacy. Distally, the antebrachiocarpal joint is flexed to facilitate a transverse arthrotomy, incising the joint capsule near the radial carpal bone. Extensor tendons are transected at the joint level, releasing the distal bone segment medially and laterally (15, 57).

The caudal aspect of the radius is dissected by rotating the fragment cranially, allowing careful inspection of the distal radial articular surface for signs of pathological fracture or tumor rupture. An incision is made in the caudal medial fascia at the level of the planned osteotomy to examine tissues caudal to the distal radius, assessing the integrity of the tumor pseudocapsule near the flexor muscles. If tumor invasion is suspected, the radial carpal bone, ulnar carpal bone, and part of the accessory carpal bone may be excised to increase the distal margin or prompt consideration of limb amputation due to heightened risk of local recurrence (15, 57). In cases of radial integrity, the arthrotomy is extended laterally to the ulna and medially to transect the medial collateral ligament and the distal insertion of the extensor carpi radialis tendon. This step ensures thorough tumor removal while preserving joint stability where possible.

The radial diaphyseal osteotomy should be transverse to maximize the bone-implant interface and is performed using an oscillating saw, aiming to preserve the caudal interosseous artery. The medial third of the ulna is ostectomized in a distal-to-proximal direction to preserve the lateral two-thirds of the distal ulna. The proximal surface of the radial and ulnar carpal bones should be leveled with an oscillating saw to enhance contact with the distal aspect of the implant. An arthrodesis can be performed by debriding the articular cartilage of the carpal bones and applying cancellous or corticocancellous bone grafts into the joint spaces. However, this procedure is currently considered unnecessary, as complete bone fusion is not required for stable limb function throughout the lifetime of most dogs with appendicular OSA (15).

### 2.2 Partial ulnectomy

**Figure 3:**
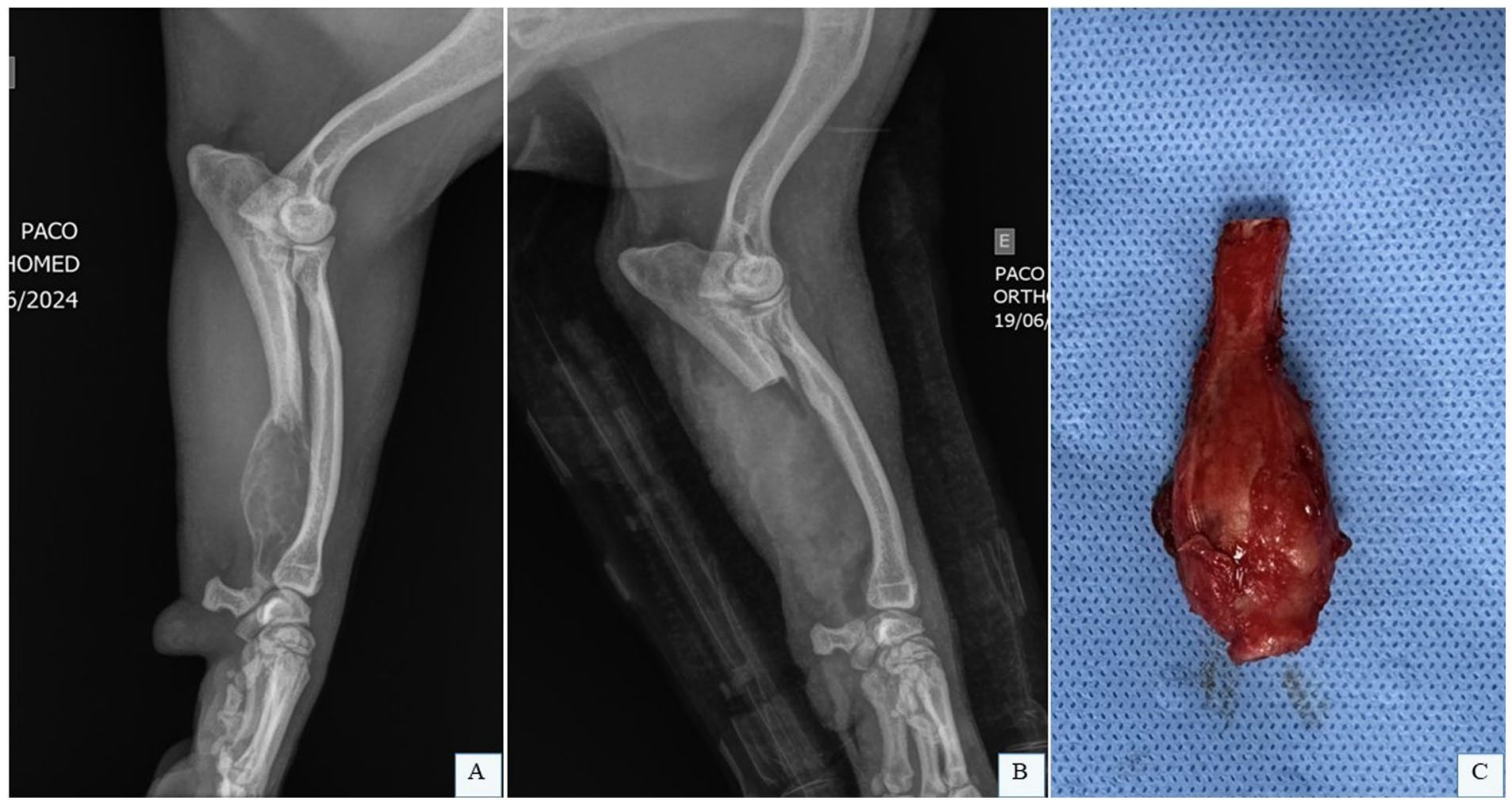
Partial ulnectomy. **(A)** Radiographic image in laterolateral projection of the left radius and ulna of a canine patient with OSA in the distal ulna. **(B)** Radiographic image in laterolateral projection of the left radius and ulna of a canine patient after the partial ulnar ostectomy. **(C)** Fragment of the distal ulna of a dog with OSA after partial ulnectomy. Dr. Renato Dornas – Small Animal Surgery Service, Universidade Federal de Minas Gerais (UFMG), Belo Horizonte, Brazil; none of the images have been published in any other scientific work.

Ulnar OSA most commonly affects the distal ulna, though other regions may also be involved (59). Bone biopsy is typically performed postoperatively, as most ulnar neoplasms are malignant, and tumor type rarely alters the surgical approach. Minimum surgical margins of 3.0 cm are recommended, which can be accessed via CT or nuclear scintigraphy. Evaluation of the integrity of the radius is mandatory; if compromised, a more extensive surgical approach is required (59). Partial ulnectomy (Figure 3) generally yields good to excellent clinical outcomes in dogs and cats, even without post-resection reconstruction or stabilization, allowing near-normal limb function; the use of this technique has recently been described (28, 29).

**Figure 4:**
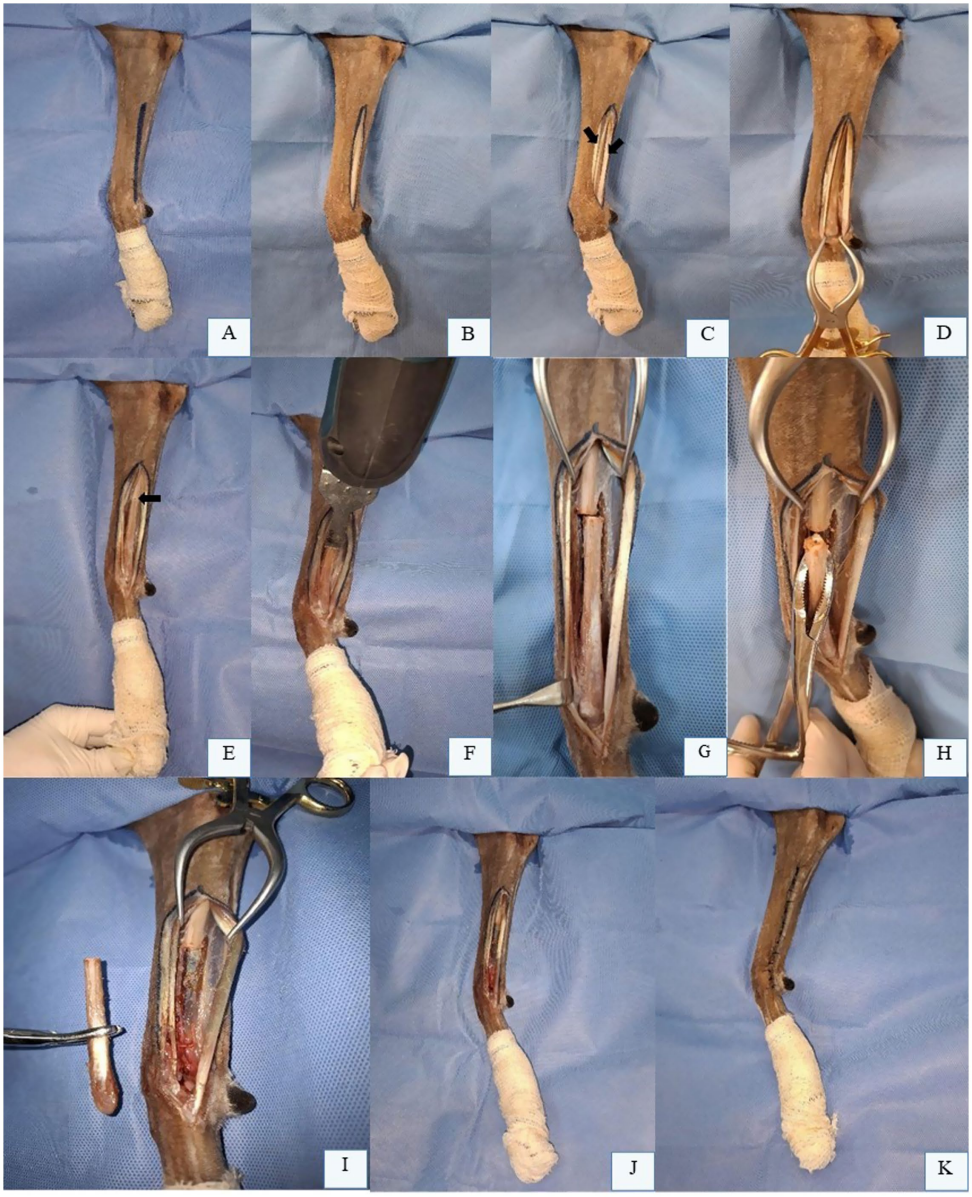
Partial Ulnectomy in the Left Thoracic Limb (LTL) of a Canine Cadaver in Right Lateral Recumbency. **(A)** Skin incision marking along the lateral aspect of the ulna, from the mid-diaphysis to the styloid process. **(B)** Subcutaneous tissue dissection. **(C)** Visualization and incision of the antebrachial fascia, with identification of the lateral digital extensor tendon (cranial arrow) and lateral ulnar tendon (caudal arrow). **(D)** Ulnar surface exposed after retraction of fascia and tendons. **(E)** Osteotomy site pre-marked with an oscillating saw (black arrow). **(F)** Ulnar diaphyseal osteotomy using an oscillating saw. **(G)** Ulna after complete cortical transection. H: Bone forceps grasping the osteotomized ulnar segment prior to distal disarticulation. **(I)** Fully removed ulnar segment. **(J)** Remaining ulna post-osteotomy, requiring no reconstruction or stabilization. **(K)** Final surgical site appearance after partial ulnectomy. Original images captured by the authors during a cadaveric surgical demonstration for academic purposes; none of the images have been published in any other scientific work.

The patient is positioned in lateral recumbency with the affected limb uppermost, clipped and aseptically prepared for partial ulnectomy. A skin incision is made along the lateral aspect of the ulna, extending from the mid-diaphysis to the styloid process (Figure 4A). Subcutaneous tissues are dissected (Figure 4B), and the antebrachial fascia is identified and incised (Figure 4C). The tendons of the lateral digital extensor muscle (cranially) and lateral ulnar muscle (caudally) are visualized and retracted to expose the ulnar surface. For enhanced exposure, the origin of the *abductor pollicis longus* muscle may be partially elevated from the cranial border of the distal ulna (Figure 4D). The approach can be extended distally to expose the carpal and metacarpal bon es if required. After confirming adequate margins (black arrow, Figure 4E), osteotomy is performed using an oscillating saw under continuous saline irrigation (Figures 4F,G). The ulnar fragment is removed with bone forceps (Figure 4H) and disarticulated from the carpus (Figure 4I). Intraoperative assessment of radiocarpal joint stability is critical following excision of the lateral styloid process. In most dogs, reconstruction of the bony column is unnecessary (Figure 4J). Closure involves suturing the antebrachial fascia, subcutaneous tissue, and skin in layers (Figure 4K) (28, 59).

### 2.3 Cortical allograft

The massive cortical allograft for distal radius reconstruction was the pioneering technique for limb-sparing surgery in dogs, also referred to as the traditional limb-sparing approach. However, despite various proposed modifications, it has fallen out of favor due to its associated disadvantages (57). The main limitations of the cortical allograft include a high infection rate, reported to be approximately 50%, and the lack of incorporation of the large grafted bone segment into the host tissue. This predisposes the graft to significant bone sequestration and persistent infections. Additionally, mechanical implant failure is a frequent complication, exacerbated by the lack of adequate revascularization of the graft (15). Other factors contributing to the obsolescence of this technique include the need for a bone bank, which is not always available, and the prolonged intraoperative preparation of the allograft (15).

The distal articular surface of the graft should be sectioned to create a flat contact interface, and the proximal osteotomy is tailored to match the radial defect length. The medullary cavity is enlarged with a curette and filled with polymethylmethacrylate (PMMA). PMMA injection enhances screw pullout strength, significantly reinforces allograft stability, and reduces complications such as screw loosening or allograft fracture (30). Additionally, incorporating heat-stable antibiotics into the PMMA is recommended to achieve sustained local antibiotic release, maintaining high concentrations over extended periods. The orthopedic implant, which is typically a plate, is temporarily secured to the cortical allograft using distal and proximal/central screws. After removal of these screws, the intramedullary canal of the cortical allograft is fully filled with PMMA (30).

As the PMMA begins to harden, the cortical bone screws are reinserted to secure the bone plate to the cortical allograft. The allograft-plate construct is then positioned within the defect, and screws are applied under compression to achieve stable apposition between the allograft and proximal radius. Additional screws are placed into the radial carpal bone and third metacarpal bone to enhance fixation. A supplementary orthogonal plate applied medially is recommended to minimize mechanical complications, with at least 80% of the metacarpal bone covered by the plate to reduce fracture risk (31). This dual-plate configuration optimizes load distribution and structural integrity, particularly in weight-bearing reconstructions.

### 2.4 Endoprosthesis

Endoprostheses, developed as an alternative to cortical allografts, offer several advantages (32). Currently, there is a growing interest in the clinical application of endoprostheses in dogs (10). A biomechanical study comparing allografts and endoprostheses in cadaver limbs demonstrated that endoprostheses exhibit superior mechanical stability (32). Furthermore, the modes of failure differ between these implants: allografts typically fail at the distal screws, while endoprostheses fail at the proximal screws (32).

**Figure 5:**
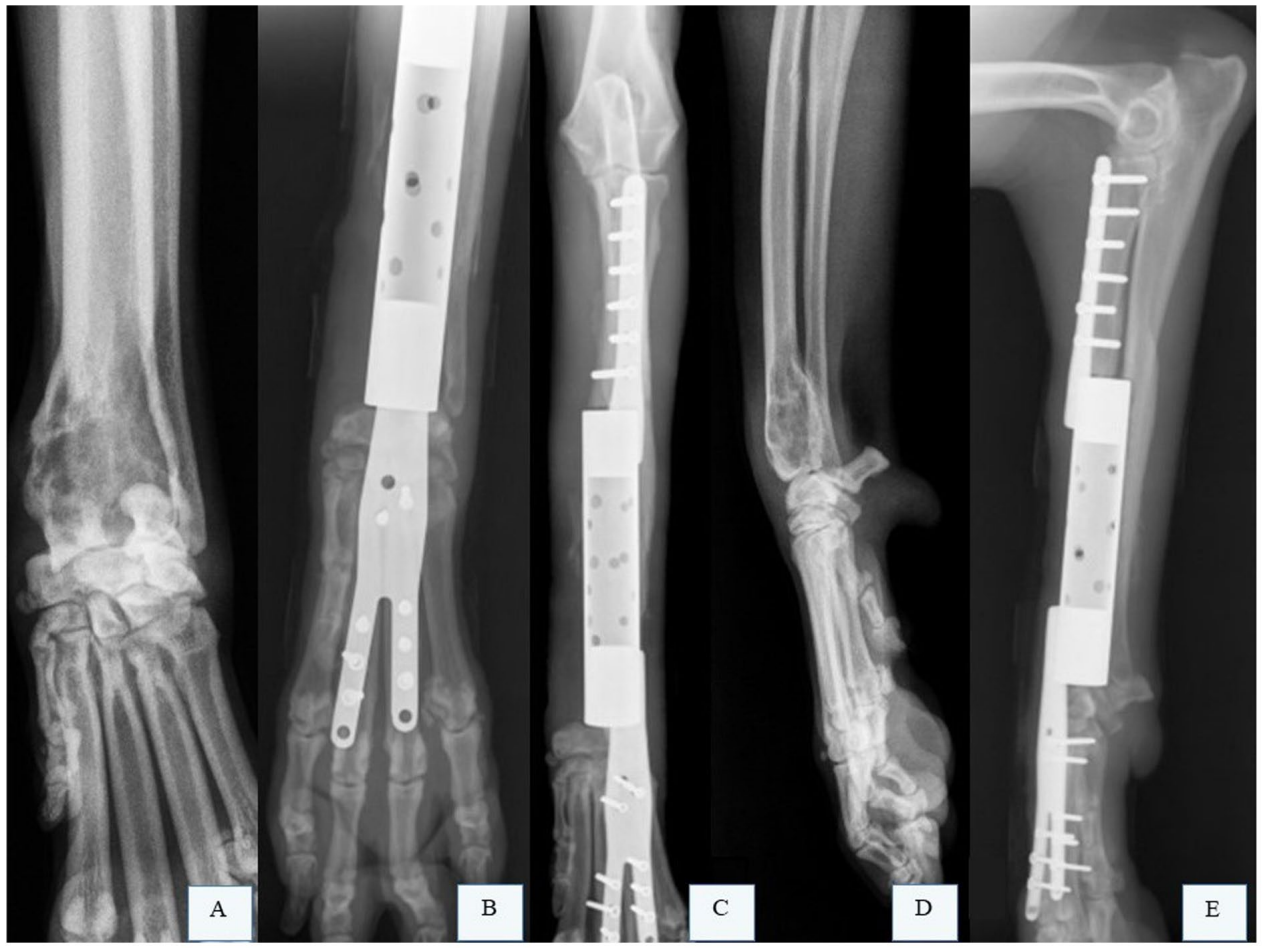
Radiographic images of the right radius and ulna of a canine patient with osteosarcoma (OSA) that underwent limb preservation surgery and reconstruction with a patient-specific titanium endoprosthesis. **(A)** Radiographic image in the craniocaudal projection of the right radius and ulna of a canine patient with OSA in the distal radius. **(B,C)** Radiographic images of the right radius and ulna after limb-sparing surgery with partial ulnar resection and reconstruction using a patient-specific titanium endoprosthesis. **(D)** Radiographic image in the mediolateral projection of the right radius and ulna of a canine patient with OSA in the distal radius. **(E)** Radiographic image of the right radius and ulna after limb-sparing surgery with partial ulnar resection and reconstruction using a patient-specific titanium endoprosthesis. Courtesy of Dr. Eduardo Capasso dos Anjos Afonso; none of the images have been published in any other scientific work.

Commercially available endoprosthesis may be combined with cortical allografts, providing customizable solutions tailored to anatomical requirements (5, 12). Recent reports of the use of this technique with patient-specific designs, enabled by computer-aided modeling and 3D printing, further enhance precision in complex cases (Figure 5) (10, 25, 33). Key benefits include reduced operative time and immediate availability, streamlining surgical workflows (5, 32, 33). However, limitations such as standardized sizing may necessitate intraoperative modifications, potentially prolonging surgery and increasing infection risks (34).

Séguin et al. (33) reported a series of five dogs with appendicular OSA (OSA) treated using tailored prostheses designed for individual patients. These implants optimized mechanical load distribution and anatomical fit, potentially reducing surgical time and complication risks. Similarly, Timercan et al. (34) utilized finite element analysis to create patient-specific implants and cutting guides, achieving a 25–50% reduction in operative duration. However, the primary limitation of this approach is the extended time required for implant design and 3D printing. Notably, all dogs in Séguin et al. (33) study developed surgical site infections, underscoring the need for stringent aseptic protocols despite the technique’s biomechanical advantages.

Following resection of the radius and ulna with appropriate safety margins, the carpal joint is prepared for arthrodesis and subsequent implant application. Typically, the proximal radial osteotomy is performed based on tumor location, with the radius excised at the level of its distal articulation. However, Wustefeld-Janssens, Lafferty, and Séguin (18) described a modified technique in which the distal osteotomy is conducted below the carpal joint, traversing the four main metacarpal bones, to address tumor involvement of the carpal joint. This adaptation ensures wider distal margins while preserving functional alignment.

During limb-sparing prosthesis application, some surgeons bend the limb-sparing plate at a 10° angle at the carpal joint, while others maintain a straight configuration (5, 32). The plate is then coupled to the prosthesis, and the construct is positioned within the defect. Care is taken to maximize contact between the implant and bone surfaces, ensuring no gaps remain distally at the radial carpal bone or proximally at the radius. Gaps significantly increase mechanical stress and implant failure risk (15).

Following proper implant positioning, the bone plate is secured to the host bone. Distally, the plate is typically anchored to the third metacarpal bone; however, the fourth metacarpal may be used to optimize paw rotation if required. For large-breed dogs, 3.5 mm cortical bone screws are used in the proximal radius and radial carpal bone, while 2.7 mm cortical screws are employed in the metacarpal bone (5). For giant breeds, 4.5 mm cortical screws are recommended proximally in the radius and 3.5 mm screws distally in the metacarpal, ensuring the plate covers at least 80% of the metacarpal length (5). The pronator, supinator, and extensor muscles are apposed using absorbable monofilament suture. Subcutaneous tissues and skin are closed routinely, with careful attention to cutaneous edge alignment and preservation of the cephalic vein. Radiographs are assessed to verify implant placement, bone-implant contact integrity, distal screw alignment in the third or fourth metacarpal, and adequate plate coverage over the metacarpal (5, 31).

Postoperative management may include placing a closed suction drain at the proximal elbow region (5), typically removed 24 h postoperatively. Additionally, biomaterials such as calcium sulfate impregnated with antibiotics can be applied to the surgical site to reduce infection risk. The limb is protected with a padded bandage, changed daily until incisional drainage ceases.

In a retrospective study of 45 dogs undergoing distal radial limb-sparing procedures with prosthesis reconstruction, 95% experienced complications (17). The most frequent was surgical site infection (78%), with Staphylococcus spp., Pseudomonas spp., *Escherichia coli*, and Enterobacter spp. commonly isolated. Implant-related complications occurred in 36% of cases, local tumor recurrence in 24, and 20% required secondary amputation (median time of 125 days) due to complications (17). Loss of deep nociception in thoracic limb digits, likely due to inadvertent neurectomy during tumor dissection, has also been reported (18).

Various limb-sparing alternatives to cortical allografts have been described over the years. The initial approach remains consistent, involving en bloc resection of the osseous tumor and adjacent tissues with adequate margins. However, techniques for reconstructing the subsequent radial defect differ significantly. Each method prioritizes balancing oncological efficacy with functional preservation, tailored to tumor location, patient size, and biomechanical demands (5, 25, 33).

### 2.5 Treated autograft

Autograft reconstruction of the distal radius is a well-established limb-sparing technique, utilizing methods such as autoclaving, pasteurization (11, 35, 36), and more recently, liquid nitrogen treatment (37). Developed as an alternative to cortical allografts, autografts eliminate the need for bone banks and enhance bone consolidation rates, albeit with increased surgical time.

**Figure 6:**
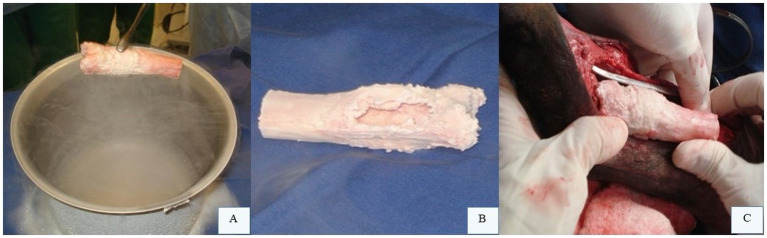
Intraoperative bone allograft treatment. **(A)** Liquid nitrogen application. **(B)** Excised tumor-bearing bone segment treated with liquid nitrogen to eradicate neoplastic cells. **(C)** Treated bone segment repositioned into the surgical defect for plate fixation. Courtesy of Dr. Arthur Gouveia Rocha – Small Animal Surgery Service, Universidade Estadual Paulista (UNESP), Jaboticabal, Brazil; none of the images have been published in any other scientific work.

Pasteurization involves submerging the tumor-bearing bone in a sterile water bath at 65 °C for 40 min (11, 36), while liquid nitrogen protocols (Figure 6A) employ cycles of freezing at −196 °C for 20 min, thawing at 20 °C for 15 min, and incubation in saline at 30 °C for 10 min (38). Unlike autoclaving, which uses high heat, pasteurization and liquid nitrogen preserve bone morphogenetic proteins essential for healing, enhancing consolidation rates (11, 36, 37). Following tumor resection with adequate margins, soft tissues are removed, and the bone segment undergoes sterilization via the chosen method (Figure 6B). If arthrodesis is planned, joint preparation is completed during this phase. The treated bone is then reimplanted into the defect (Figure 6C) and secured with a bone plate, mirroring cortical allograft fixation techniques (15).

Yazawa et al. (37) reported three clinical cases of dogs with appendicular OSA in the thoracic limb. Surgical management involved liquid nitrogen treatment of the autologous bone followed by reconstruction. The affected sites included the left proximal scapula, right proximal humerus, and right distal radius. After tumor resection, soft tissues were excised, and the bone segment was treated with liquid nitrogen at −196 °C, thawed at room temperature, and incubated in saline before being reimplanted and fixed with orthopedic implants. The authors noted successful postoperative weight-bearing, ease of defect reconstruction, and a lower incidence of surgical site infections compared to other methods. The technique’s simplicity, requiring no specialized equipment, was emphasized as a key advantage. Yazawa et al. (37) considered that liquid nitrogen-treated autografts are a highly effective and promising alternative to thoracic limb amputation.

Complications associated with autograft pasteurization closely resemble those observed with allografts, including local tumor recurrence, graft infection, and implant failure. However, the risk of implant failure may be marginally higher in pasteurized autografts due to pre-existing osteolytic lesions in the affected bone, which remain unaddressed by bone cement augmentation during reconstruction (15).

### 2.6 Vascularized ulnar transposition or ulnar rollover transposition

First described by Séguin et al. (31), this technique utilizes the ipsilateral distal ulna as a vascularized rollover autograft to reconstruct the radial defect following distal radial tumor excision. There are reports of its use in three dogs by Séguin et al. (31), in two dogs by Irvine-Smith and Lobetti (39), and in 26 dogs by Séguin et al. (6). All of these animals were diagnosed with distal radial OSA. During distal radial resection, meticulous attention is paid to preserving the insertions of the abductor pollicis longus and quadrate pronator muscles to the ulnar periosteum, as well as maintaining the integrity of the ulna itself. Since the distal radius is separated from the ulna during this procedure, absence of neoplastic involvement in the ulna is critical for eligibility. A key contraindication for this technique is tumors affecting >50% of the radial length, as resection exceeding 54% of the radius increases the risk of residual radial fracture in dogs undergoing ulnar roll-over (6).

Following distal radial resection with appropriate margins, the ulna is preserved with its blood supply from the caudal interosseous vessels. The muscular insertions of the *abductor pollicis longus*, ulnar head of the deep digital flexor, and quadrate pronator at the distal ulna can also be preserved. A distal ulnar osteotomy allows en bloc removal of the ulnar styloid process and distal radius. The carpus is prepared for routine arthrodesis, and the ulnar medullary cavity is debrided with a curette and filled with bone cement (6). The ulnar graft is then rotated into the radial defect. A bone plate is applied, extending from the proximal radius to the metacarpophalangeal joints, to stabilize the reconstruction (6).

The vascularized ulnar transposition technique offers significant advantages, including a vascularized autograft with high osteogenic potential, reducing infection risk and implant failure, while avoiding donor site morbidity (6).

In a study of 27 limbs treated with this method, complications were reported in 20 cases (6). Biomechanical issues predominated, with 15 cases involving complications such as fractures of the residual radius, all of which occurred in dogs where >54% of the radial length was excised. Surgical site infections arose in 12 limbs, and local tumor recurrence was observed in 2 cases. Severe complications necessitated secondary amputation in 2 dogs. Additionally, forearm shortening—a recognized drawback of the technique—averaged 15% across 7 limbs, potentially impacting gait symmetry and biomechanics. Despite these challenges, the median disease-free interval was 245 days.

### 2.7 Lateral translation of the manus

A modified technique derived from the ulnar roll-over procedure, termed lateral manus translation, has been reported in 18 dogs in one single study (16). This adaptation aims to reduce the risk of radial fractures associated with the original method by preserving the structural integrity of the ulna. Studies indicate that ulnar preservation does not significantly elevate the risk of local tumor recurrence (16, 17).

In this procedure, resection of a portion of the distal radius and part of the medial cortex of the ulna is performed as previously described, and the articular surfaces of the carpal bones are prepared for arthrodesis. The manus is translated laterally so that the distal end of the ulna contacts the radial carpal bone. A limb-sparing plate is placed dorsally from the remaining proximal radius to the third metacarpal bone, acting as a bridging implant. The authors of the technique bend the plate caudally at an angle of 10°–15° to improve contact between the manus and the distal ulnar surface. Additionally, a 3.5 mm String of Pearls plate is applied laterally, extending from the proximal ulna to the dorsal aspect of the fourth metacarpal (16).

In this study, complications were reported in 12 thoracic limbs, including infection in 10, biomechanical failure in 6, and local recurrence in 4 dogs (16). None of the dogs experienced residual radial fractures, and limb function was acceptable in all cases. The advantages of this technique include no requirement for a vascularized ulnar autograft and the ability to remove large radial segments. The primary disadvantage is its inefficacy in patients with ulnar involvement (16).

### 2.8 Bone transport osteogenesis

Bone transport osteogenesis is a specific application of distraction osteogenesis, initially developed by Russian surgeon Gavriil Ilizarov. This technique has been employed to reconstruct bone defects in the distal radius following OSA resection as one option among reconstructive techniques (55). It is worth noting that there is no recent clinical description of its use (15). It involves the progressive, gradual, and prolonged replacement of the excised distal radius with regenerated bone through the distraction of bone surfaces, while an external skeletal fixator shares mechanical loads and permits weight-bearing (40, 41). The newly formed bone tissue undergoes remodeling into lamellar bone, analogous to intramembranous ossification (27).

Among the advantages of this technique is the formation of new, healthy bone tissue that is densely vascularized, infection-resistant, biomechanically robust, and capable of rapid remodeling to support weight-bearing. Additionally, it does not require permanent implants (40, 41, 55). However, the primary disadvantage lies in the prolonged need for a circular external skeletal fixator (41). Open discussion with the owner regarding financial costs and long-term commitment is essential to ensure their informed adherence to the treatment plan, including daily distraction procedures and periodic radiographic assessments.

Patient selection criteria significantly influence treatment success. According to Ehrhart (55), ideal candidates are those with tumors involving less than 50% of the bone length and minimal soft tissue involvement. This is supported by the rationale that, following appropriate-margin excision, sufficient residual bone must remain to create a transport segment and accommodate at least three fixation pins proximal to it. Patients with pathological fractures, neoplasia in another location, metastasis, or severe comorbidities should not be considered favorable candidates (55).

The daily distraction rate is 1 mm, and the fixator remains in place until bone consolidation is achieved (41). Ehrhart (55) reported a mean distraction interval of 123 days and an average of 205 days until implant removal in nine dogs. Degna et al. (40) documented a required distraction period of 120 to 147 days for treating OSA in the distal radius of six dogs. Double bone transport osteogenesis is a proposed modification to shorten transport time by utilizing two transport segments. This technique was employed by Rovesti et al. (42) for treating OSA in the distal tibia, addressing an 11 cm defect that required 92 days of distraction and 162 days until definitive implant removal. Additionally, transverse bone transport osteogenesis has been experimentally described for limb-sparing surgery following distal radius resection. This involves longitudinal osteotomy of the ulna (41).

For this technique, after resection of the distal radius and osteotomy of a proximal radial transport segment, a circular external fixator is constructed with five rings, the central ring serving as the transport unit. Distraction of the transport segment may commence 3 to 7 days postoperatively. While three to four daily distractions are possible, two 0.5 mm increments (totaling 1 mm/day) are typically sufficient. Serial radiographs should be performed every 10 to 14 days to monitor progress (41, 55).

Following the completion of the transport period, the fixator remains in place during a consolidation phase, during which the regenerated bone gains strength and the transport segment integrates with the adjacent bone (55). The transport segment is further distracted distally for 1 to 3 days after contact with the carpal bones to achieve compression. The application of autogenous cancellous bone graft enhances the likelihood of successful consolidation. Compression of the transport segment against the adjacent bone and rigid fixation are critical to treatment success and must be achieved to facilitate healing (55).

Postoperative care requires analgesic and anti-inflammatory management, along with protective bandaging. Dogs typically begin ambulating by the second postoperative day. Serial radiographs every 10 to 14 days are recommended to monitor distraction progress. The fixator may be removed once radiographic evidence of bone consolidation is confirmed. A splint may be applied for up to 14 days following implant removal to assist in the gradual transfer of limb stress during weight-bearing (55). Reported complications include pin breakage, skin invagination at pin sites, local tumor recurrence, non-union, and lameness (40, 55).

### 2.9 Endo-exo prostheses

**Figure 7:**
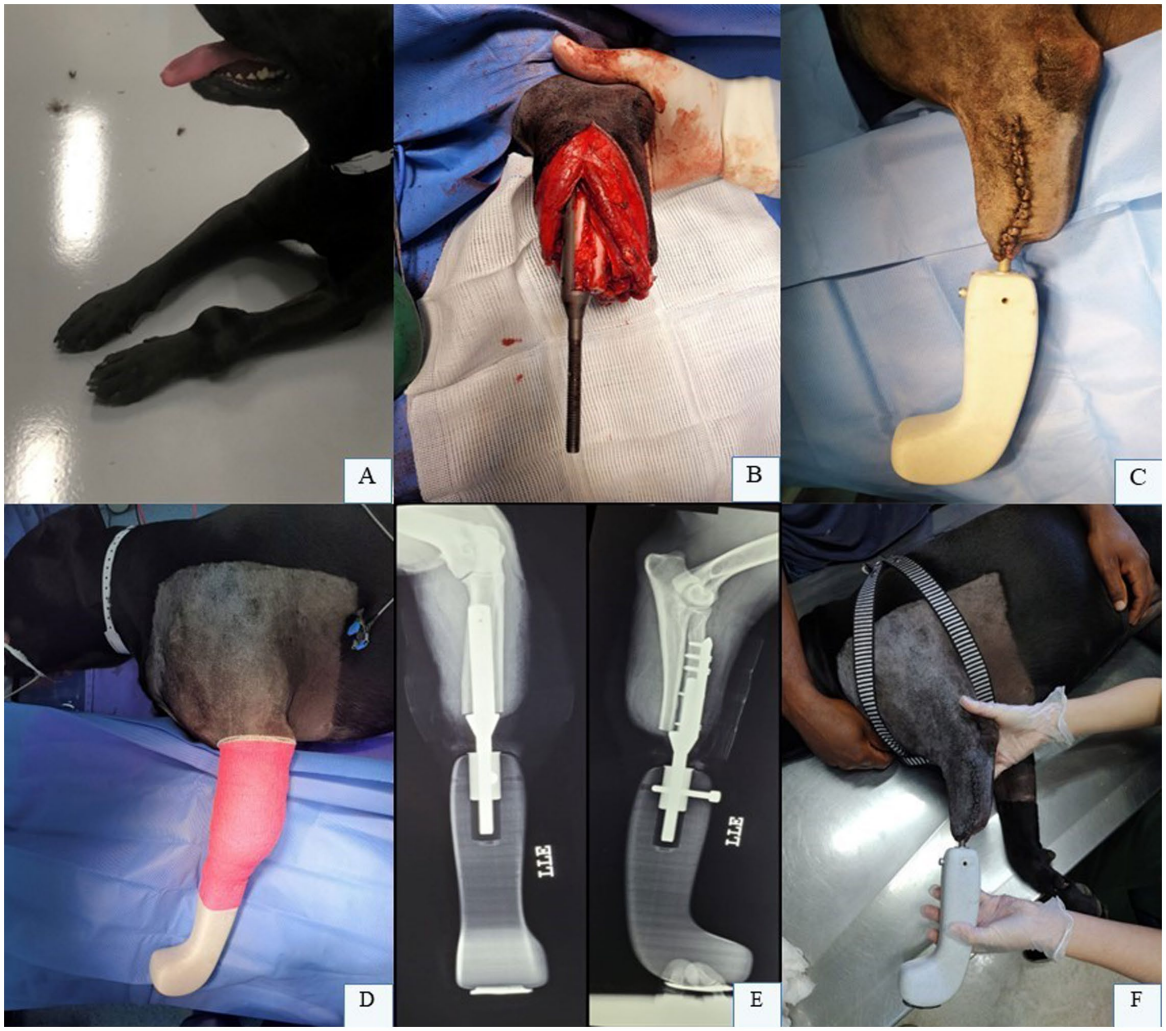
**(A)** Osteosarcoma located in the distal third of the left radius and ulna in a canine patient. **(B)** Endoprosthesis being fixed. **(C,D)** Final aspect of the endo-exoprosthesis in the left thoracic limb during the immediate postoperative period. **(E)** Radiographic images in craniocaudal and laterolateral projections of the canine forearm following partial limb amputation and implantation of the endo-exoprosthesis in the left thoracic limb. **(F)** Patient follow-up assessment performed 10 days postoperatively. Dr. Renato Dornas – Small Animal Surgery Service, Universidade Federal de Minas Gerais (UFMG), Belo Horizonte, Brazil; none of the images have been published in any other scientific work.

Endo-exo prostheses involve partial limb amputation for a distal primary bone tumor, wherein the amputated segment is replaced with a prosthesis (Figure 7) and its use is well documented (10, 43, 44). This prosthesis includes a prosthetic foot attached to either an intramedullary or extramedullary implant (10, 43, 44). These devices are custom-manufactured for the patient following meticulous surgical planning, which encompasses material selection, prosthesis design, and alignment with the contralateral limb based on patient-specific requirements. Successful implementation of this technique depends critically on osseointegration and soft tissue integration (10, 43).

These prostheses should be designed with the objectives of improving quality of life, preventing the progression or development of degenerative joint disease, achieving homogeneous limb length, and enabling routine physical, rehabilitation, and functional activities (10). Three-dimensional scanners are utilized to capture and analyze the limb’s topography, while advanced imaging modalities such as CT scans reconstruct the remaining osseous structures. Three-dimensional computational systems then facilitate the biomechanical design of the prosthesis with high precision. The prostheses are fabricated using metal additive manufacturing technologies (10, 34).

Canine limb exoprostheses frequently comprise four components: a socket, a liner, a suspension system, and a shock-absorbing pylon. The socket serves as the primary element of the exoprosthesis; its fits on the residual limb must facilitate effective load transmission, stability, and comfort. The liner, integrated with the suspension system, should be soft and comfortable, while the suspension system ensures secure positioning of the socket. The shock-absorbing pylon, constructed from stainless steel, titanium, or aluminum, is located at the distal extremity of the exoprosthesis and mitigates shock forces generated during high-impact activities (10).

The design of prosthetic feet poses significant challenges, often necessitating multiple design iterations to achieve an optimal functional outcome (10). Steel and titanium are frequently utilized materials for intramedullary or extramedullary implants. Titanium is a preferred choice due to its biocompatibility, lightweight properties, and elastic modulus comparable to that of bone, which minimizes stress shielding (10, 34, 43).

Socket-type prostheses rely on the limb-socket interface for force transmission and fixation, a design that in human medicine has been linked to frequent complications such as friction-induced ulcers, infection, and tissue necrosis (10, 43, 44). A prospective study involving 12 dogs undergoing partial limb amputation with socket exoprostheses reported universal complications, including challenges in device retention, pressure ulcers, latent surgical site infections, and prosthesis aversion (45).

The complications can be mitigated through the use of transcutaneous prostheses (44), a type of endo-exo prosthesis comprising an intramedullary or extramedullary endoprosthesis, an inert percutaneous device, and distally, the exoprosthesis. In this design, mechanical loads are transferred from the exoprosthesis to the bone via the endoprosthesis (10, 44). Potential complications associated with transcutaneous implants include cutaneous irritation and infection, skin breakdown, prosthesis fixation failure, and gradual extrusion of the percutaneous apparatus secondary to inadequate epithelial growth or cutaneous marsupialization (43).

Drygas et al. (43) described a case of a dog with bilateral partial tibial amputation treated with an endo-exoprosthesis. The patient ambulated with assistance in the immediate postoperative period and independently within 7 days, with no complications. At 14 months, the right tibial implant underwent aseptic loosening and was replaced with a newly fabricated implant. At 17 months post-revision and 26 months after the initial procedure, the dog exhibited normal activity levels. The authors concluded that transcutaneous tibial implants are potentially viable for functional restoration of pelvic limbs in amputee dogs.

Fitzpatrick et al. (44) reported the treatment of four dogs with malignant bone neoplasia in the distal radius (3 dogs) and tibia (1 dog). All patients regained limb function post-surgery, demonstrated favorable osseointegration, and ambulated without pain for 8 to 18 months postoperatively. Cyclic implant failure due to fracture at the distal bone-implant interface was described in one patient with radial involvement. The authors highlighted the success of the treatment, noting that the skin-implant interfaces were robust, resilient, and free of regression, infection, or cutaneous marsupialization around the external implant component. Postoperative care included supportive bandaging during the initial days, appropriate analgesia and antibiotic therapy, as well as physical rehabilitation and periodic radiographic monitoring (44).

### 2.10 Microwave ablation

Microwave ablation is a non-surgical limb-sparing technique in which microwaves generate an electromagnetic field that forces the oscillation and rotation of polar molecules, such as water, at a frequency of two to five billion cycles per minute (46). This technique was recently introduced into the literature by a pioneering clinical pilot study conducted by Salyer et al. (46) and reported as part of the multimodal treatment of OSA in dogs (47). The resulting increase in kinetic energy generates heat, which is distributed uniformly through the tissue, effectively killing tumor cells within the ablation zone. This process achieves temperatures exceeding 55 °C, inducing acute coagulative necrosis (46).

The local effects of microwave ablation were investigated in six dogs with OSA of the distal radius. Following a dorsal or medial approach to the antebrachium and lateral retraction of the common digital extensor tendon, a 2 mm Steinmann pin was introduced to create a bone tunnel within the tumor (for subsequent ablation probe placement), extending from the distal epiphysis to the proximal diaphysis of the lesion. The tunnel was centered between the metaphysis and diaphysis. Temperatures were maintained between 45 °C and 55 °C, and the probe was repositioned multiple times to treat the entire lesion via overlapping ablation zones based on presurgical planning. No intraoperative or peri-procedural complications were reported up to 48 h post-ablation, after which limb amputation was performed (46). Microwave-induced tumor necrosis ranged from 30 to 90%, with a median of 55%. The results of this study suggest that microwave ablation is a feasible limb-sparing technique in dogs.

### 2.11 Shortening of the limb

This technique was first recently described by Boston and Skinner (48), who also reported its use in a single case of a canine patient with distal radial OSA. The procedure consisted of resection of the bone tumor with arthrodesis of the remaining radius to the carpus. This limb-sparing technique aims to shorten the limb and may be combined with an endoprosthesis or exoprosthesis, thereby avoiding complications commonly associated with reconstructive limb-preservation methods. A notable advantage is the elimination of the need to reconstruct the distal radial defect (48). The authors recommend that selection criteria include canine patients with distal radial OSA showing no evidence of severe metastatic disease in bone or lungs, lesions confined within cortical bone with minimal soft tissue involvement, and proximal margins of 2–3 cm beyond the cranial extent of the osseous lesion. Additionally, limb shortening should not exceed 20% to preserve functional outcomes (48).

In the reported case, a craniomedial approach to the radius was performed. The radius and ulna were transversely osteotomized at the same level, and the proximal articular surface of the radial carpal bone was prepared for arthrodesis and apposed to the remaining distal radius. A hybrid 2.7/3.5 mm locking carpal arthrodesis plate was applied to the radius and third metacarpal bone. Reported complications included a focal area of dermal necrosis with subsequent infection, a fracture of the third metacarpal bone through a screw hole, and limb length discrepancy. Following management of these complications, the patient demonstrated satisfactory ambulation (48).

### 2.12 Stereotatic radiosurgery

Stereotactic radiosurgery of the tumour was described by Farese et al. (60) as a technique based on the precise delivery of high-dose radiation to the tumor over a shortened treatment period, with minimal radiation exposure to adjacent tissues. This approach has gained increasing use as a minimally invasive limb-sparing option for appendicular OSA (49, 50). It offers the advantage of avoiding surgical procedures and eliminating the need for permanent implants and risk of infection. However, stereotactic radiosurgery remains limited in availability and carries significant risks of pathological fracture, infection, and implant failure (14, 15, 35, 51).

In its initial implementation, 1.6 mm Kirschner wires were inserted around the affected bone, distal to the neoplasm, to create fiducial markers within the target region. These fiducial markers aid in delineating areas for radiation treatment or surgical intervention, ensuring precise spatial alignment. The patient then underwent contrast-enhanced CT for radiation dose planning. Following this, the patient was transferred to the radiation suite, where a single dose of 3,000 cGy was administered, after which the pins were removed (60).

Farese et al. (60) reported this procedure in 11 dogs, including 9 with OSA of the distal radius, 1 of the distal ulna, and 1 of the distal tibia. Pathological fractures developed in four dogs, likely due to a combination of neoplastic lytic effects and radiation-induced osteonecrosis. Local recurrence occurred in three cases, potentially attributable to the use of CT rather than MRI for planning, which offers lower soft tissue resolution. Adverse effects such as alopecia, desquamation, and cutaneous hyperpigmentation were documented. The authors concluded that this technique is most suitable for small neoplasms with limited osseous destruction (60).

Covey et al. (51) evaluated the clinical outcomes of six dogs with appendicular OSA treated with stereotactic radiosurgery followed by internal fixation of pathological fractures in a retrospective study. Two dogs presented with pathological fractures at initial evaluation, while the remaining four developed fractures after stereotactic radiosurgery. One fracture was managed with an external skeletal fixator, and five were stabilized with plates, three of which were placed using minimally invasive techniques. Deep tissue infections were diagnosed in five patients, with *Pseudomonas aeruginosa*, *Staphylococcus* spp., *Klebsiella pneumoniae*, *Escherichia coli*, and *Aeromonas hydrophila* isolated as causative agents. Implant failure occurred in three patients, two of whom underwent surgical revision and one of whom required limb amputation. Radiographic evidence of bone union was not observed at any point during postoperative monitoring. Despite the high complication rate, the authors subjectively assessed limb function as good when implants remained stable and infections were absent or subclinical. They concluded that fracture management via internal fixation remains a viable alternative for pathological fractures following stereotactic radiosurgery (51).

Contrary to this assertion, Boston et al. (35) conducted a retrospective study of 18 dogs with appendicular OSA treated with stereotactic radiosurgery, adjuvant chemotherapy, and stabilization of pathological fractures. Complications occurred in 16/17 dogs, with 15 classified as severe. The most frequent complication was infection, observed in 15 dogs, two of which progressed to sepsis. Two dogs exhibited radiographic and cytological evidence of tumor progression, and one dog underwent amputation. Nine dogs required amputation due to infection or fracture at a median of 152 days post-initial stabilization. The mean survival time was 344 days. Based on these findings, the authors do not recommend stereotactic radiotherapy with concurrent stabilization in dogs with OSA at high risk for or presenting with pathological fractures due to the high complication rate. They instead propose that alternative limb-sparing procedures or amputation should be prioritized in such cases (35).

Martin et al. (50) conducted a multi-institutional retrospective study of 123 dogs undergoing stereotactic radiotherapy for the treatment of appendicular OSA. Gait assessment data were available for 98 monitored dogs, with 82 demonstrating improved lameness at a median of 3 weeks post-treatment. Among dogs monitored for fractures (51/125), 41% developed pathological fractures at a median of 106 days following stereotactic radiotherapy, and 21% underwent subsequent amputation. The median survival time was 233 days for dogs treated with stereotactic radiotherapy and 346 days for those that received limb amputation. The authors propose that stereotactic radiosurgery combined with chemotherapy represents a viable non-surgical alternative for limb preservation in carefully selected patients (50).

## 3 Postoperative care and complications in reconstructive surgeries

Postoperative care requires tailored management strategies depending on the surgical technique employed. Pain control should ideally follow a multimodal approach, combining traditional opioids with nonsteroidal anti-inflammatory drugs (NSAIDs). Systemic broad-spectrum antibiotics are indicated due to vascular compromise, the presence of implants, and the high rate of postoperative infections reported in the literature. Seroma and edema may be mitigated through the application of a Robert Jones bandage; however, digits should be monitored periodically for swelling, as venous and lymphatic drainage is frequently impaired following reconstructive procedures. Patients are typically ambulatory within 12 to 24 h postoperatively, with rest and a gradual reintroduction of light physical activity—such as short leash walks—recommended. Serial radiographs of the affected limb and thorax are essential for early detection of complications and assessment of metastatic disease (15, 57).

Complication rates in reconstructive limb-sparing techniques are notably high, exceeding those associated with amputations and other orthopedic procedures (13). The most prevalent complications include infection, local recurrence, and implant failure. In a retrospective study of 192 dogs undergoing distal radial limb-sparing surgery with reconstruction via allograft, metallic endoprosthesis, or vascularized ipsilateral ulnar graft, complications were reported in 80% of cases (13). Surgical site infections occurred in 62% of these patients, with 85 bacterial species identified; *Enterococcus faecalis*, *Escherichia coli*, *Pseudomonas aeruginosa*, and *Staphylococcus pseudintermedius* were the most frequently isolated pathogens. Mechanical failure was observed in 40% of patients, and local tumor recurrence occurred in 22%. Secondary amputation due to complications was required in 16% (31/192) of dogs included in the study (13).

The reported infection rate for limb-sparing surgical procedures varies widely, ranging from 40 to 78% (6, 13, 17, 32, 52). The severity of these infections spans from localized surgical wound infections to severe deep tissue involvement, such as osteomyelitis. Risk factors associated with infections and management challenges include insufficient soft tissue coverage around allografts or implants, poor vascularization in distal radial and ulnar regions, extensive surgical dissection, immunosuppression secondary to neoplasia or chemotherapy (16, 17, 52).

Several studies report an association between surgical site infection and prolonged survival in patients undergoing limb-sparing procedures (13, 52), a correlation not observed in amputees (53). The proposed theory posits that chronic infection may exert immunomodulatory effects on cytotoxic cells and macrophages, potentially stimulating the synthesis of antiangiogenic factors (13, 52). In contrast, for amputees, the transient nature of surgical site infections may limit the duration required to elicit such innate immune responses (53).

Local recurrence rates were reported at 24% in a study of 28 dogs undergoing limb-sparing surgery with endoprosthesis placement (17). This risk escalates if the tumor capsule is inadvertently ruptured intraoperatively. Preoperative imaging modalities such as CT and MRI can enhance surgical planning by evaluating ulnar involvement and soft tissue extension, thereby reducing the likelihood of inadequate surgical margins. Local recurrence is most commonly managed with limb amputation following thorough restaging of the patient (13).

Mechanical implant failure occurred in 42% of canine patients treated with endoprostheses and in 92% of limbs reconstructed with cortical bone grafts (5). Similarly, a 36% failure rate was observed in dogs receiving endoprostheses (17). In the former study, endoprosthesis failures were noted to occur proximally, whereas allograft failures were localized distally (5). Distraction osteogenesis is susceptible to fixation pin failure, which is managed through pin replacement. Severe or catastrophic failures may necessitate surgical revision or amputation (13).

The selection of a specific technique depends on the preference of the surgeon, expertise, and available equipment. Each method carries distinct advantages and disadvantages. Regrettably, the overall complication rate for limb-sparing procedures in dogs remains high. Ongoing efforts are required to refine surgical protocols and develop optimal techniques for canine OSA patients, as well as other species.

### 3.1 Cementoplasty

Cementoplasty, a minimally invasive and technically straightforward procedure, involves the injection of a bone substitute and serves as an exclusive palliative treatment option for pain relief, prevention of pathological fractures, and improvement in quality of life for patients whose owners decline amputation. This technique does not influence disease progression or patient survival (47, 54). It may be combined with microwave ablation to enhance osseous stability. Calcium phosphate-based bone substitutes are commercially available and have been documented for use in the distal radius, proximal humerus, proximal tibia, and metacarpal bones of dogs with OSA (47, 54).

Following careful surgical access to preserve adjacent soft tissues and joints, along with microwave ablation or tumor content aspiration using a surgical suction device, a Jamshidi needle is positioned through intact cortical bone into the tumor site to minimize additional tissue injury and tumor cell dissemination. The cavity is then slowly filled with bone cement or calcium phosphate-based material. Radiography, fluoroscopy, or CT is employed intra- and postoperatively to assess material distribution and detect potential cement leakage (47, 54).

Improvements in pain and lameness have been reported. Postoperative care should include appropriate analgesia, restricted physical activity, and soft bandaging (54). Complications such as deep infection and pathological fractures were documented in a study of 13 dogs with thoracic limb OSA treated with calcium phosphate-based bone substitutes (54). Additional reported adverse events include intra-articular cement leakage, venous thrombosis, and pathological fractures (47).

## 4 Conclusion

The surgical management of canine appendicular OSA necessitates a multifaceted, patient-tailored approach. Critical factors include meticulous preoperative planning, alignment of tumor biological behavior with surgical strategy, and precise anatomical execution to preserve critical structures. While limb-sparing techniques offer functional preservation, their success hinges on balancing oncologic control with complication mitigation. Optimal outcomes depend on integrating evidence-based techniques and rigorous patient selection, underscoring the surgical role aimed at extending survival time while prioritizing quality of life.
